# Collateral Chemoresistance to Anti-Microtubule Agents in a Lung Cancer Cell Line with Acquired Resistance to Erlotinib

**DOI:** 10.1371/journal.pone.0123901

**Published:** 2015-04-14

**Authors:** Hiroshi Mizuuchi, Kenichi Suda, Katsuaki Sato, Shuta Tomida, Yoshihiko Fujita, Yoshihisa Kobayashi, Yoshihiko Maehara, Yoshitaka Sekido, Kazuto Nishio, Tetsuya Mitsudomi

**Affiliations:** 1 Division of Thoracic Surgery, Department of Surgery, Kinki University Faculty of Medicine, Osaka-Sayama, Japan; 2 Department of Surgery and Science, Graduate School of Medical Sciences, Kyushu University, Fukuoka, Japan; 3 Department of Genome Biology, Kinki University Faculty of Medicine, Osaka-Sayama, Japan; 4 Division of Molecular Oncology, Aichi Cancer Center Research Institute, Nagoya, Japan; Institute of Medical Science, University of Tokyo, JAPAN

## Abstract

Various alterations underlying acquired resistance to epidermal growth factor receptor-tyrosine kinase inhibitors (EGFR-TKIs) have been described. Although treatment strategies specific for these mechanisms are under development, cytotoxic agents are currently employed to treat many patients following failure of EGFR-TKIs. However, the effect of TKI resistance on sensitivity to these cytotoxic agents is mostly unclear. This study investigated the sensitivity of erlotinib-resistant tumor cells to five cytotoxic agents using an *in vitro* EGFR-TKI-resistant model. Four erlotinib-sensitive lung adenocarcinoma cell lines and their resistant derivatives were tested. Of the resistant cell lines, all but one showed a similar sensitivity to the tested drugs as their parental cells. HCC4006ER cells with epithelial mesenchymal transition features acquired resistance to the three microtubule-targeting agents, docetaxel, paclitaxel and vinorelbine, but not to cisplatin and gemcitabine. Gene expression array and immunoblotting demonstrated that ATP-binding cassette subfamily B, member 1 (*ABCB1*) was up-regulated in HCC4006ER cells. *ABCB1* knockdown by siRNA partially restored sensitivity to the anti-microtubule agents but not to erlotinib. Moreover, the histone deacetylase inhibitor entinostat sensitized HCC4006ER cells to anti-microtubule agents through ABCB1 suppression. Our study indicates that sensitivity of tumor cells to cytotoxic agents in general does not change before and after failure of EGFR-TKIs. However, we describe that two different molecular alterations confer acquired resistance to EGFR-TKIs and cytotoxic agents, respectively. This phenomenon should be kept in mind in selection of subsequent therapy after failure of EGFR-TKIs.

## Introduction

Adenocarcinoma is the most common histological subtype of lung cancer, and somatic mutation of the epidermal growth factor receptor (*EGFR*) is present in approximately 40% and 20% of these tumors in East-Asians and Caucasians, respectively [1]. Treatment of lung adenocarcinoma patients with EGFR tyrosine kinase inhibitors (TKIs) prolongs progression-free survival compared with conventional cytotoxic chemotherapy [2–5]. However, the development of acquired resistance to EGFR-TKIs is almost inevitable. Many molecular or histological aberrations underlying this acquired resistance have been reported, including *EGFR* T790M secondary mutation, *MET* amplification, *ERBB2* amplification, hepatocyte growth factor overexpression, epithelial to mesenchymal transition (EMT), and small cell lung cancer transformation [6,7].

Strategies to cope with acquired resistance to EGFR-TKIs that are based on each different resistant mechanism would be ideal, and such approaches are currently being developed. However, in current clinical practice, these patients are typically treated with cytotoxic chemotherapeutic agents, selection of which is often empirical. It is also unclear whether acquired resistance to EGFR-TKIs affects sensitivity to cytotoxic drugs.

In this study, we evaluate the growth inhibitory effects of these cytotoxic drugs by comparing cells resistant to an EGFR-TKI with their parent cells using an *in vitro* model. Isogenic resistant clones derived from parental cells have a common genetic background, and this resistance model is able to be used to evaluate the influence of different resistant mechanisms on chemosensitivity.

## Materials and Methods

### Cell lines and reagents

The human lung adenocarcinoma cell lines HCC827, HCC4006 and H358 were kind gifts from Dr AF Gazdar (Hamon Center for Therapeutic Oncology Research, University of Texas Southwestern Medical Center at Dallas). These cell lines have been commonly used in *in vitro* experiments [8–13]. PC9 cells were kindly provided from Dr K Nishio (Department of Genome Biology, Kinki University Faculty of Medicine). This cell line has also been commonly used in previous researches elsewhere [14,15]. Acquired resistant cell lines established from these cells, PC9/ZD cells and HCC827TRB10 cells, were kindly provided from Dr K Nishio and Dr K Furugaki (Chugai Pharmaceutical Co., Ltd.), respectively [15,16]. HCC827ER, HCC827EPR, HCC4006ER and H358ER were established in our previous work [9,10,17]. Table 1 provides a summary of the mutational status and sensitivity to erlotinib of these cell lines [9,10,15–17]. Cells were cultured in RPMI1640 medium supplemented with 10% heat-inactivated fetal bovine serum (FBS) at 37°C in a humidified incubator with 5% CO_2_. Cisplatin (CDDP), gemcitabine (GEM), docetaxel (DOC), paclitaxel (PAC), vinorelbine (VNR), erlotinib, and entinostat were purchased from Selleck Chemicals (Houston, TX).

**Table 1:** The characteristics of EGFR-TKI sensitive cell lines and their resistant clones.

Cell lines	Driver	Resistant mechanisms	Erlotinib IC50(μM)
HCC827	*EGFR* del19	-	0.0065
HCC827ER^(8)^		*MET* amplification	6.9
HCC827EPR^(8)^		T790M	7.1
HCC827TRB10^(12)^		loss of amplified *EGFR*	5.7
HCC4006	*EGFR* del19	-	0.030
HCC4006ER^(9)^		EMT	>10
PC9	*EGFR* del19	-	0.023
PC9/ZD^(11)^		T790M	2.2
H358	*KRAS* G12C	-	0.12
H358ER^(10)^		IGF1R hyperactivation	3.3

**Abbreviations: EMT**, epithelial mesenchymal transition.

### Growth inhibition assay

Cell viability was measured using a Cell Counting Kit-8 (Dojindo Laboratories, Kumamoto, Japan) as previously described [17]. Briefly, 3 × 10^3^ cells (2 × 10^3^ cells for HCC827TRB10) were plated into each well of 96-well flat-bottomed plates and grown in RPMI-1640 containing 10% FBS. After 24 hours, dimethyl sulfoxide (DMSO), CDDP, GEM, DOC, PAC, VNR, and erlotinib with or without entinostat were added at the indicated drug concentration, and cells were incubated for an additional 72 hours. A colorimetric assay was performed after addition of 10 μl Cell Counting Kit-8 reagent to each well, and the plates were incubated at 37°C for 2–4 hours. Absorbance at 450nm was read using a multiplate reader (Tecan, Männedorf, Switzerland). Percent growth was expressed relative to DMSO-treated controls.

### RNA isolation and gene expression array analysis

Gene expression array analyses were carried out to assess differences between HCC4006 and HCC4006ER cells as previously described [18]. Briefly, cells were cultured without erlotinib until subconfluency. After an 8 hour-exposure to 2 μM erlotinib, total RNA was isolated using mirVana miRNA Isolation Kit (Qiagen, Venlo, the Netherlands). RNA (100 ng) from each sample was processed for hybridization using GeneChip Human Genome U133 Plus 2.0 Array (Affymetrix, Santa Clara, CA). After hybridization, the chips were processed using a High-Resolution Microarray Scanner Genechip Scanner 3000 7G (Affymetrix). The Robust Multichip Averaging (RMA) procedure was performed for normalization using the open-source R programming environment.

### Antibodies and western blot analysis

Anti-E-cadherin, anti-ATP-binding cassette subfamily B, member 1 (ABCB1), anti-class III beta-tubulin (TUBB3) and anti-beta-actin antibodies were purchased from Cell Signaling Technology (Beverly, MA).

Preparation of total cell lysates and immunoblotting was performed as previously described [17]. Cells were cultured without erlotinib until subconfluency, and media was changed to RPMI with 10% FBS containing DMSO or 1 μM entinostat. After 72 hours, cells were rinsed with phosphate-buffered saline (PBS), lysed in sodium dodecyl sulfate (SDS) buffer and homogenized. Approximately 30 μg of total cell lysate protein was subjected to SDS polyacrylamide gel electrophoresis and transferred to polyvinylidene difluoride membranes (Bio-Rad, Hercules, CA). After blocking with 2.5% nonfat dry milk and 2.5% bovine serum albumin in PBS, membranes were incubated with primary antibodies (1:1000) overnight, washed with PBS, reacted with secondary antibody (1:1000), treated with ECL solution (GE Healthcare, Fairfield, CT). Chemiluminescence was detected by EOS Kiss X6i (Canon, Tokyo, Japan). Expression values of TUBB3 relative to beta-actin were determined using Just TLC software (Sweday, Lund, Sweden).

### Preparation of cell block and immunohistochemistry

Cells grown to subconfluency were trypsinized, centrifuged and fixed with 15% neutral buffered formalin for 3 hours. Following centrifugal separation and removal of solution, the alginate sodium containing pellet was turned into a gel by drop of calcium chloride. These gels were embedded in paraffin.

Sections (4 μm) were deparaffinized and heat-induced epitope retrieval was performed with Target Retrieval Solution, pH 9 (Dako, Carpinteria, CA). Slides were treated with 3% hydrogen peroxide for 30 min and then incubated with a primary anti-ABCB1 (P-glycoprotein) antibody (1:100, Dako) overnight. Immunoreactions were detected using the Envision+ System-HRP (Dako) according to manufacturer’s protocol. The reactions were visualized followed by counter staining with hematoxylin.

### Reverse-transfection of small-interfering RNAs

Cells were reverse-transfected with 10 nM small interfering RNAs (siRNAs) mixed with Lipofectamine RNAiMAX (Invitrogen, Carlsbad, CA). The validated siRNAs specific for *ABCB1* (*ABCB1*-1, 5′-CGAUACAUGGUUUUCCGAU-3′; *ABCB1*-2, 5′-GUUUGUCUACAGUUCGUAA-3′), *CDH1* (5′-CGUAUACCCUGGUGGUUCA-3′) and nonspecific siRNAs were purchased from Applied Biosystems (Foster City, CA). Twenty-four hours after reverse-transfection, the indicated drugs were added, and cell viability was measured using the Cell Counting Kit 8 after an additional 72 hours.

## Results

### All cell lines with acquired resistance to erlotinib except HCC4006ER demonstrated similar sensitivity to cytotoxic drugs compared with their parental cells

**Fig 1:**
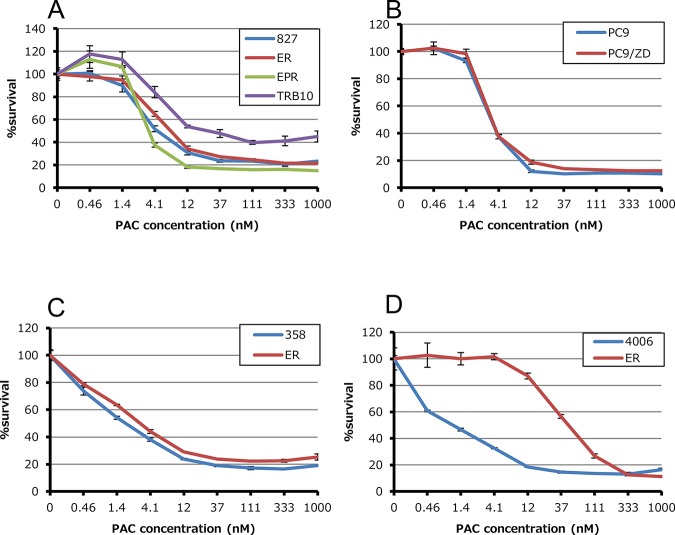
Anti-proliferative effects of paclitaxel in EGFR-TKI sensitive and their resistant clones. Tumor cells (2–3×10^3^ cells per well) were incubated with various concentrations of paclitaxel for 72 hours. Percent growth relative to DMSO-treated controls was determined by Cell Counting Kit-8 assay.

We first studied the sensitivity of cell lines to five cytotoxic agents that are commonly used in the treatment of NSCLC. CDDP, GEM, DOC, PAC and VNR sensitivity was assessed in HCC827, HCC4006, PC9 and H358 cells, as well as their EGFR-TKI resistant derivatives. In HCC827 cells and their three EGFR-TKI resistant clones (HCC827ER, HCC827EPR and HCC827TRB10), we failed to detect a difference in sensitivity to any of the five cytotoxic agents (Fig 1 and Table 2). This was also case with PC9, H358 and their resistant clones. On the other hand, in HCC4006ER cells, IC_50_ values for DOC, PAC and VNR were increased by approximately 41-, 43-, 28-fold, respectively (Fig 1 and Table 2). All three drugs to which cells showed decreased sensitivity (DOC, PAC and VNR) were anti-microtubule drugs, acting either through inhibition of microtubule polymerization (vinca alkaloids) or by depolymerization (taxanes).

**Table 2:** IC50 values for cytotoxic agents in EGFR-TKI sensitive and their resistant clones.

Cell lines	CDDP (μM)	GEM (nM)	DOC (nM)	PTX (nM)	VNR (nM)
HCC827	136	50	0.90	4.5	3.3
HCC827ER	65	15	2.0	7.1	3.5
HCC827EPR	57	63	0.91	3.4	2.3
HCC827TRB10	120	28	3.1	24.0	6.1
HCC4006	84	>10,000	<0.46	1.1	1.2
HCC4006ER	49	>10,000	19	47	33
PC9	140	15	0.64	3.2	8.7
PC9/ZD	170	26	0.48	3.3	8.6
H358	500	<4.6	0.64	1.8	1.5
H358ER	530	6.5	0.97	2.9	1.5

**Abbreviations: CDDP**, cisplatin; **GEM**, gemcitabine; **DOC**, docetaxel; **PTX**, paclitaxel; **VNR**, vinorelbine.

### ABCB1 overexpression induces insensitivity to anti-microtubule drugs in HCC4006ER cells

HCC4006ER cells were established by stepwise exposure to erlotinib (from 20 nM to 2 μM) over 4 months in our previous work [10]. To explore the molecular alterations underlying acquired resistance to anti-microtubule agents, we performed a gene expression array analysis comparing HCC4006ER cells with parental HCC4006 cells. Expression of *CDH1* in HCC4006ER cells was significantly decreased compared with HCC4006 cells, confirming the previous report (Table 3) [10]. Additionally, we observed increased expression of *ZEB1* and *ZEB2* which both regulate EMT by 27- and 23-fold, respectively. On the other hand, stem cell markers such as *OCT4*, *SOX2*, *NANOG* and *GATA4* and cancer stem cell (CSC)–like markers including *ALDH1A1*, *CD44* and *CD133* were not upregulated (Table 4).

**Table 3:** Top 30 genes altering expression between HCC4006 and HCC4006ER cells.

Upregulated genes in HCC4006ER cells compared with HCC4006 cells				Downregulated genes in HCC4006ER cells compared with HCC4006 cells			
Gene Symbol	log_2_ (ratio)	Gene Symbol	log_2_ (ratio)	Gene Symbol	log_2_ (ratio)	Gene Symbol	log_2_ (ratio)
ODZ2	8.67	ADAMTS1	5.27	CEACAM6	-10.71	COL4A3	-6.60
COL8A1	7.92	PADI2	5.06	**CDH1**	**-9.03**	HS6ST2	-6.51
SFRP2	6.80	ABCB1/ABCB4	5.05	TMC5	-8.77	FXYD3	-6.48
RGS4	6.69	ALPK2	4.99	CEACAM6	-8.38	LCN2	-6.37
NNMT	6.55	NEFM	4.98	TMC5	-8.38	HS6ST2	-6.33
244567_at	6.48	LAMA4	4.98	AGR2	-8.34	C15orf48	-6.31
GAS1	6.41	STON1	4.96	TMC5	-7.89	RAB25	-6.21
GNG4	6.28	238512_at	4.96	TMEM30B	-7.49	PTGS2	-6.20
PDE1A	6.20	C14orf132	4.93	ESRP1	-7.42	C4orf19	-6.19
BEX1	6.11	SLC16A1	4.90	MUC20	-7.29	CDH3	-6.19
CNN1	6.02	CNRIP1	4.89	PCDH20	-7.18	ITGB6	-6.18
GNG4	5.92	PAPPA	4.86	SPOCK2	-7.12	HOPX	-6.10
PTX3	5.86	NRK	4.83	TSPAN8	-7.12	NTM	-6.06
FAM101B	5.86	THBS2	4.81	C1orf116	-7.11	RNASE1	-6.05
GNG4	5.86	PDE1A	4.79	KRT19	-7.07	DLL1	-6.04
1561064_a_at	5.67	ZEB1	4.78	FGF13	-6.89	PIGR	-5.95
COL11A1	5.63	LOC100288985	4.76	TMC5	-6.86	EHF	-5.93
HMCN1	5.63	ADAMTS5	4.76	MAL2	-6.79	MUC20	-5.86
SHISA2	5.60	SLC16A1	4.69	NPNT	-6.78	STEAP4	-5.84
ADAMTS2	5.58	CLDN11	4.69	GPR87	-6.71	SCNN1A	-5.78
SLC16A1	5.42	RNF182	4.63	MPZL2	-6.71	CEACAM5	-5.73
PAX6	5.42	ADAMTS5	4.61	SCEL	-6.68	COL4A4	-5.73
AK5	5.38	COL12A1	4.61	GALNT3	-6.65	EHF	-5.71
**ABCB1**	**5.32**	DCDC2	4.60	C4orf19	-6.63	NEBL	-5.71
NNMT	5.32	NXF3	4.60	SPINK1	-6.60	LRRN1	-5.64

**Table 4:** Relative expression levels of stem cell /cancer stem cell (CSC)-like markers in HCC4006ER cells compared with HCC4006.

Type	Gene Symbol	log_2_ (ratio)
stem cell	POU5F1 (OCT4)	0.17
	SOX2	0.14
	NANOG	0.08
	GATA4	0.50
CSC-like	ALDH1A1	-0.88
	CD44	-1.11
	PROM1(CD133)	-2.37

**Fig 2:**
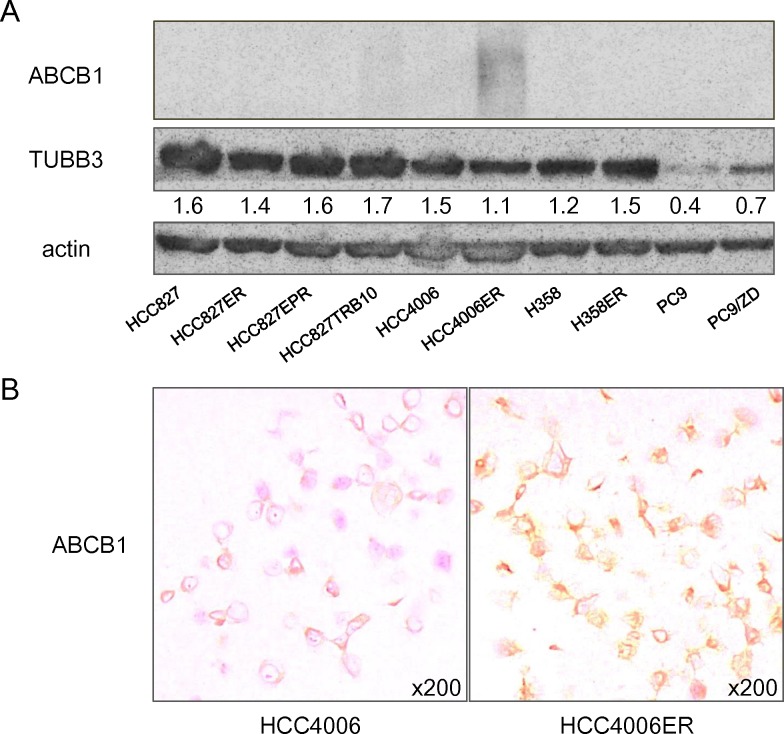
Expression of proteins which were reported to be associated with sensitivity to anti-microtubule agents. (A) Protein expression was evaluated by western blot analysis. Expression values of class Ⅲ beta-tubulin (TUBB3) relative to beta-actin were determined using Just TLC software. (B) Representative images of HCC4006 and HCC4006ER cells immunohistochemically stained with antibodies to ATP-binding cassette subfamily B, member 1 (ABCB1).

Amongst the top 50 up-regulated and 50 down-regulated genes in HCC4006ER cells compared with HCC4006 parental cells (Table 2), we identified that ATP-binding cassette subfamily B, member 1 (*ABCB1*) was up-regulated by approximately 40-fold in HCC4006ER cells. On the other hand, no other ABC transporter family member was overexpressed to a similar extent in HCC4006ER cells (Table 5). Overexpression of ABCB1, a drug efflux pump has been reported to confer inherent or acquired resistance to anti-microtubule drugs [19–24]. Immunoblotting and immunohistochemistry of cell blocks also confirmed the overexpression of ABCB1 protein in HCC4006ER cells but not in parental HCC4006 cells (Fig 2).

**Table 5:** Relative expression levels of ABC transporter family in HCC4006ER cells compared with HCC406.

Gene Symbol	log_2_ (ratio)	Gene Symbol	log_2_ (ratio)
ABCA1	0.46	ABCC12	0.38
ABCA12	-4.17	ABCC13	0.23
ABCA13	-2.68	ABCC2	-0.38
ABCA2	0.37	ABCC3	-1.84
ABCA3	-0.45	ABCC4	2.02
ABCA4	-0.46	ABCC5	0.51
ABCA5	1.84	ABCC6	-0.80
ABCA6	0.05	ABCC8	0.56
ABCA7	-0.95	ABCC9	-0.21
ABCA8	-0.68	ABCD1	0.26
ABCA9	-0.33	ABCD2	0.04
**ABCB1**	**5.32**	ABCD3	0.90
ABCB10	-0.12	ABCD4	0.39
ABCB11	0.37	ABCE1	1.15
ABCB4	-0.26	ABCF1	0.64
ABCB5	0.54	ABCF2	0.65
ABCB6	0.26	ABCF3	-0.10
ABCB7	-0.51	ABCG1	-1.73
ABCB8	-0.03	ABCG2	-0.66
ABCB9	0.37	ABCG4	-0.16
ABCC1	-0.77	ABCG5	0.00
ABCC10	-0.86	ABCG8	0.35
ABCC11	0.47		

Moreover, increased expression of class Ⅲ beta-tubulin (TUBB3) has also been reported to be a predictive marker for clinical outcome of taxane/vinorelbine-based chemotherapy [25]. However, immunoblotting demonstrated that the expression level of TUBB3 in HCC4006ER cells was not altered (Fig 2). Gene expression array also revealed that alteration of TUBB3 was less than 2 fold (data not shown). Since it is reported that docetaxel-resistant cell line expressed ~50-fold more TUBB3 than parental cells [26], we assumed that the contribution of TUBB3 in our system was minimal.

**Fig 3:**
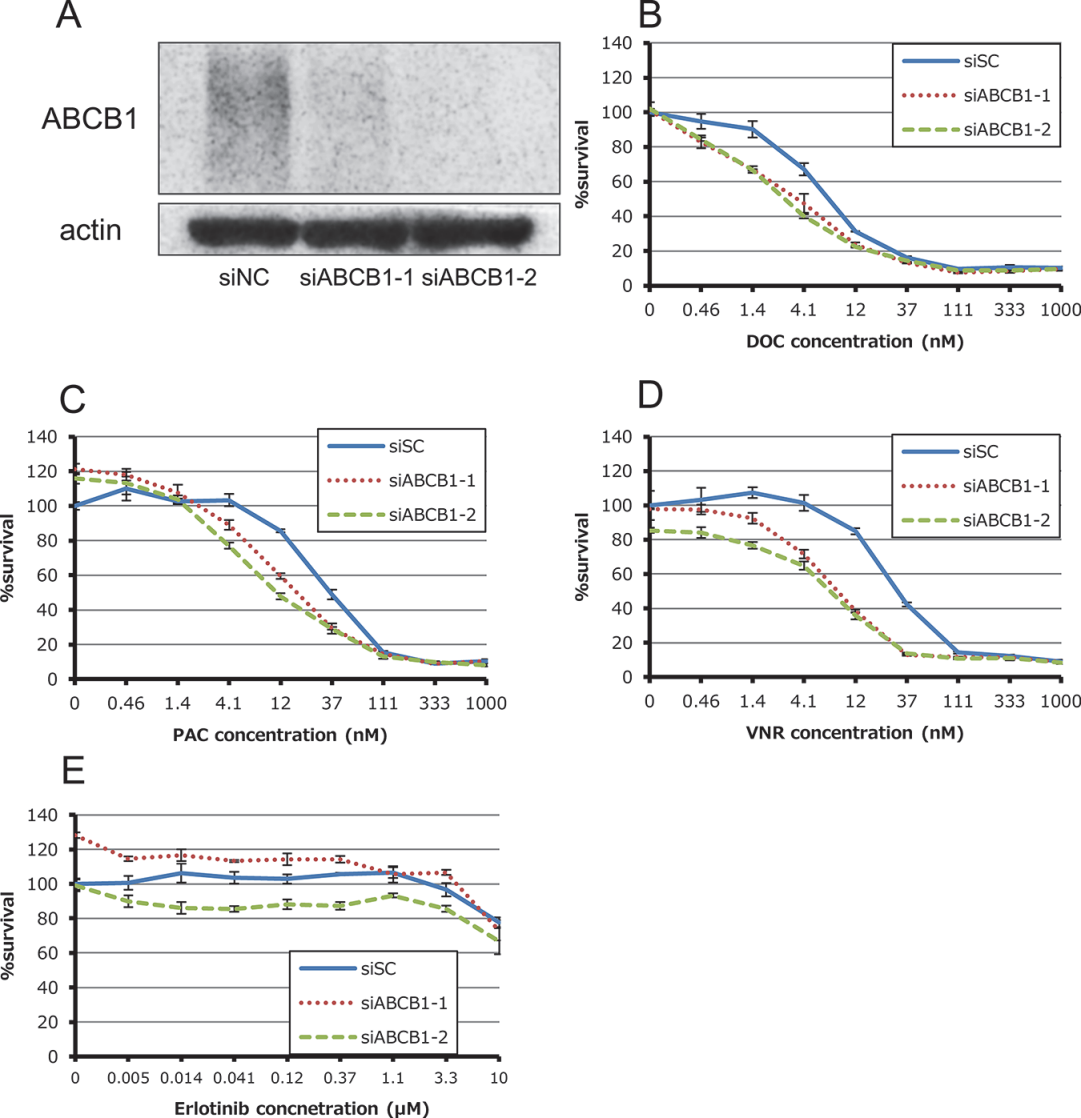
Effect of siRNA-mediated knockdown for ABCB1 in HCC4006ER cells. (A) Total cell lysates were harvested 72 hours after reverse-transfection of negative control siRNA (siNC) or validated two siRNAs for ATP-binding cassette subfamily B, member 1 (ABCB1) mixed with Lipofectamine RNAiMAX. (B-E) Tumor cells were reverse-transfected at the same time plating into 96-wells and then incubated for 24 hours. They were incubated with various concentrations of docetaxel, paclitaxel, vinorelbine and erlotinib for additional 72 hours. Percent growth relative to DMSO-treated controls was evaluated by Cell Counting Kit-8 assay.

We next evaluated the role of overexpressed ABCB1 in acquired resistance to anti-microtubule drugs by using two validated siRNAs for *ABCB1*. siRNA-mediated knockdown of *ABCB1* partially sensitized HCC4006ER cells to anti-microtubule agents (Fig 3). In contrast, *ABCB1* depletion did not restore erlotinib sensitivity in HCC4006ER cells (Fig 3).

**Fig 4:**
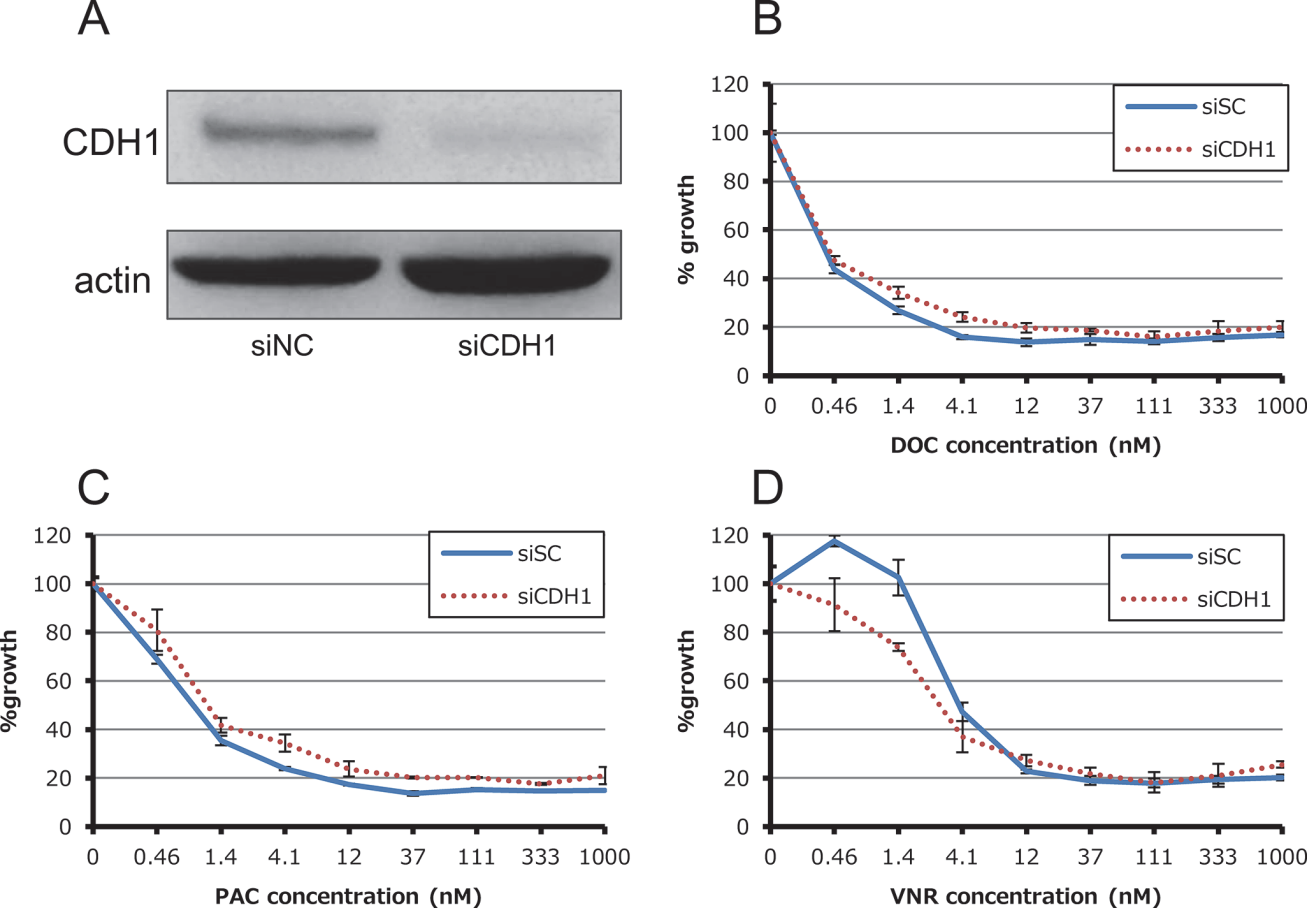
Effect of siRNA-mediated knockdown for CDH1 in HCC4006ER cells. (A) Total cell lysates were harvested 72 hours after reverse-transfection of negative control siRNA (siNC) or validated siRNAs for CDH1 which encodes E-cadherin mixed with Lipofectamine RNAiMAX. (B-D) Tumor cells were reverse-transfected at the same time plating into 96-wells and then incubated for 24 hours. They were incubated with various concentrations of docetaxel, paclitaxel and vinorelbine for additional 72 hours. Percent growth relative to DMSO-treated controls was evaluated by Cell Counting Kit-8 assay.

We also investigated whether loss of E-cadherin activity influences sensitivity to anti-microtubule agents. Knockdown of *CDH1* (gene encoding E-cadherin) in HCC4006 cells did not affect sensitivity to any of the three anti-microtubule agents (Fig 4).

### Entinostat alleviates resistance to anti-microtubule drugs via suppression of ABCB1 in HCC4006ER cells

**Fig 5:**
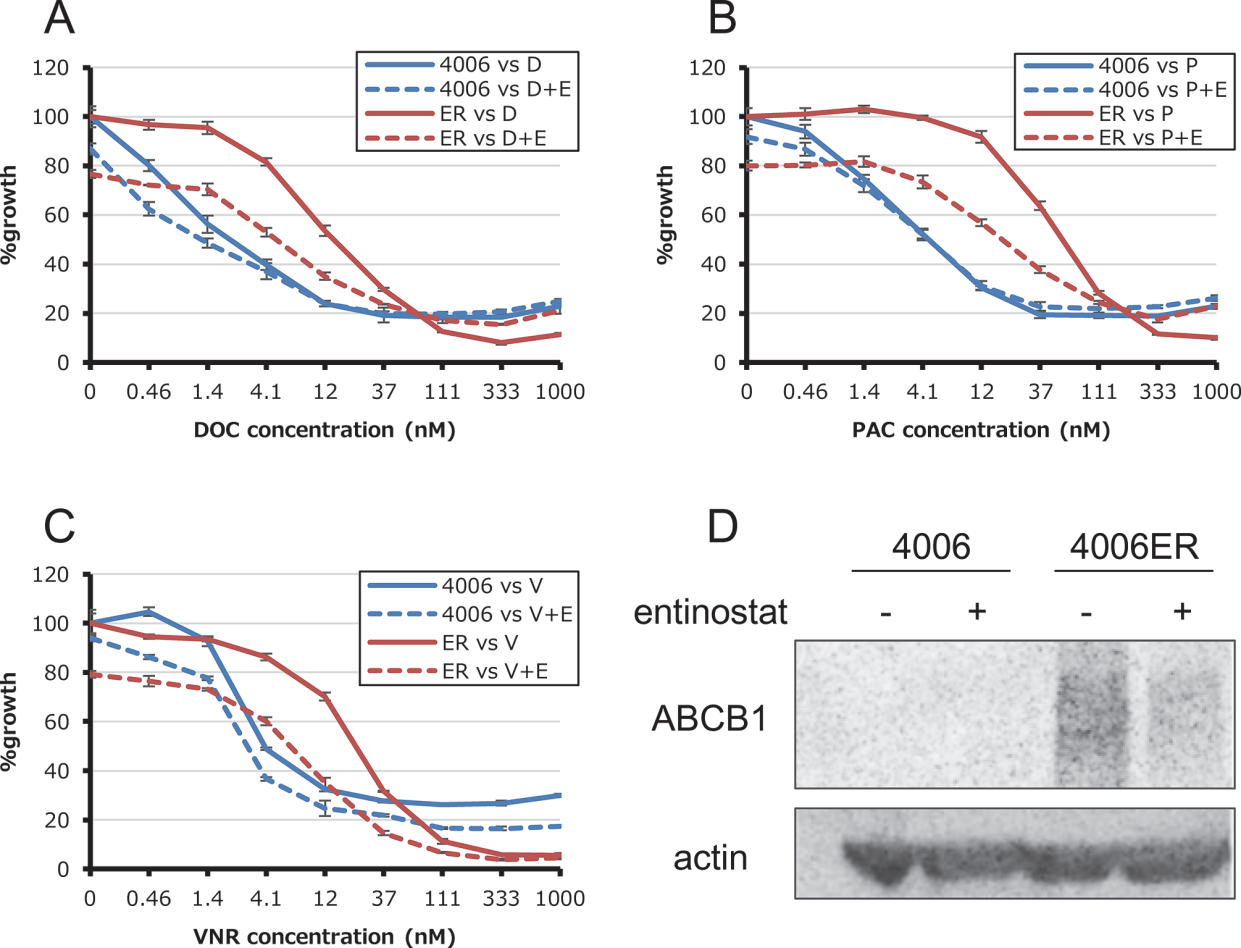
Additional effects of entinostat in HCC4006 and HCC4006ER cells. (A-C) Tumor cells were incubated with various concentrations of docetaxel (D), paclitaxel (P) and vinorelbine (V) and with or without 1μM entinostat (E) for 72 hours. Percent growth relative to DMSO-treated controls was evaluated by Cell Counting Kit-8 assay. (D) Total cell lysates were harvested after addition of 1μM entinostat.

We next treated HCC4006ER cells with the class I histone deacetylase (HDAC) inhibitor entinostat, which reverses EMT in HCC4006ER cells and restores sensitivity to erlotinib as described in our previous study [10]. Here, entinostat did not alter sensitivity to anti-microtubule agents in HCC4006 cells (Fig 5). However, this agent restored sensitivity to anti-microtubule drugs in HCC4006ER cells (Fig 5). To explore the molecular mechanism which entinostat achieves this, we examined the expression levels of ABCB1 in HCC4006ER cells with or without exposure to 1 μM entinostat. Treatment with entinostat markedly reduced the expression of ABCB1 protein after 72 hours (Fig 5).

## Discussion

**Fig 6:**
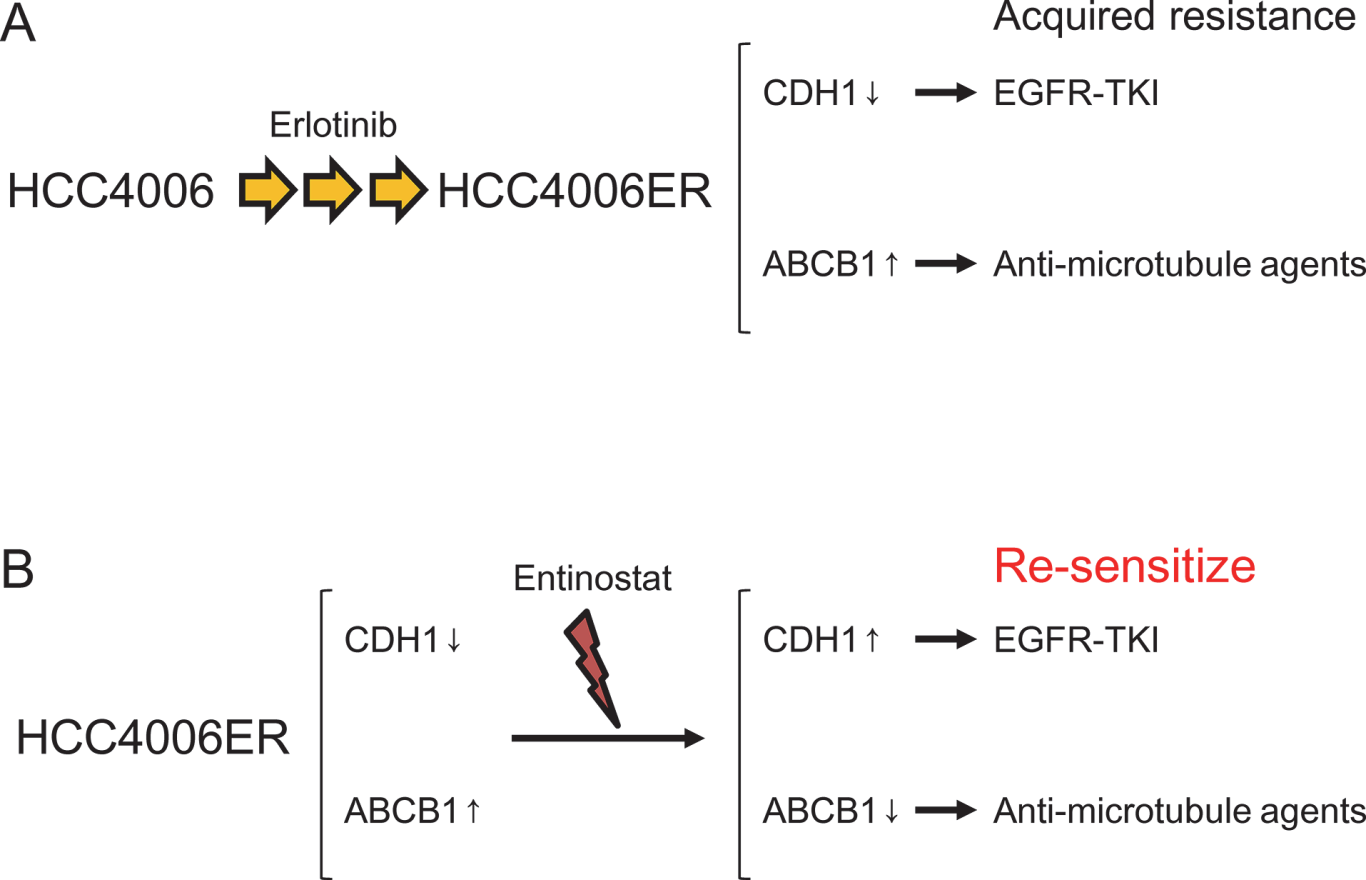
Diagram for the proposed mechanisms of cross-resistance to EGFR-TKIs and anti-microtubule agents. (A) HCC4006ER cells which were established by chronic exposure to erlotinib lose expression of E-cadherin and overexpress ABCB1. These alterations confer acquired resistance to EGFR-TKIs and anti-microtubule agents, respectively. (B) A histone deacytylase inhibitor entinostat sensitizes HCC4006ER cells to EGFR-TKIs and anti-microtubule agents via re-expression of E-cadherin and suppression of ABCB1, respectively.

We have previously shown that HCC4006ER cells are resistant to erlotinib through acquisition of EMT as characterized by the down-regulation of E-cadherin expression [10]. In the present study, we observed that HCC4006 ER cells have a similar sensitivity to CDDP and GEM compared with their parental cell line, but are more resistant to anti-microtubule agents. ABCB1 overexpression in HCC4006ER cells was responsible for this phenomenon. However, restoration of sensitivity to anti-microtubule agents by ABCB1 siRNA was not complete. This may be due to involvement of other genes than ABCB1 which was suggested by expression profiling or incomplete suppression of ABCB1 gene expression in our system (Table 3). On the other hand, ABCB1 expression was not related to sensitivity to erlotinib. In fact, Noguchi et al. previously reported that ABCB1 induction in two lung cancer cell lines did not change sensitivity to erlotinib [27]. ABC transporters including ABCB1 have been reported to be implicated in promoting cancer stem cell (CSC)-like properties [28]. Although our HCC4006ER cells did not show increased expression of the other CSC-like markers (Table 5), Shien et al. have recently reported similar observations that gefitinib-resistant HCC827 cells exhibiting both EMT features and CSC properties with overexpression of ABCB1 were resistant to DOC and PAC [29]. Therefore, this “collateral” cross-resistance to erlotinib and anti-microtubule agents resulted from two distinct mechanisms, both of which were thought to be a cause of or result from EMT (Fig 6).

Alternatively, there are several examples in which resistance to EGFR-TKI and chemotherapeutic agents shared the same molecular mechanism. PTEN deficiency renders PC9 cells resistant to cisplatin and also reduces their sensitivity to erlotinib [30]. AXL overexpression confers resistance to gefitinib and cisplatin in HCC4006 and HCC827 cells [31]. These mechanisms have also been reported to induce resistance to EGFR-TKIs [32,33]. Therefore, they can be regarded as “shared” cross-resistance to EGFR-TKI and cytotoxic drugs, in contrast to the “collateral” resistance described here.

We also found that the class I HDAC inhibitor entinostat restored sensitivity to anti-microtubule drugs through down-regulation of ABCB1 protein. We have previously shown that entinostat restores sensitivity to erlotinib by promoting E-cadherin re-expression [10]. These observations suggest that the EMT phenotype and ABCB1 overexpression observed in HCC4006ER cells was at least partly attributable to histone deacetylation. Therefore, HDAC inhibition might be an attractive approach to combine with EGFR-TKI to delay or suppress the emergence of resistance.

In contrast to the above, five cell lines with acquired resistance to erlotinib, (two by T790M secondary mutations, one each by *MET* amplification, loss of amplified *EGFR* and IGF1R hyperactivation) did not exhibit any detectable change in sensitivity to five cytotoxic agents. This is consistent with previous *in vitro* observations that demonstrated that EGFR-TKI resistant cells with T790M or *MET* amplification showed sensitivity to cytotoxic agents similar to that of their parental cells [14,29]. These alterations are considered to account for more than 60–70% of cases of acquired resistance to EGFR-TKIs [7]. Therefore, it can be hypothesized that sensitivity to cytotoxic agents does not change before or after EGFR-TKI treatment and vice versa in the majority of cases. This hypothesis is consistent with the finding that overall survival of the patients treated with platinum-doublet chemotherapy as the first line treatment is not significantly different from that of patients treated with front-line EGFR-TKI, provided that the cross-over was high enough in patients with lung cancer harboring EGFR mutation [2,3].

In conclusion, we have demonstrated that erlotinib resitstance in a cell line harboring *EGFR* mutation conferred a “collateral” resistance to anti-microtubule agents via upregulation of *ABCB1*. However, this phenomenon appeared exceptional and chemosensitivity was not influenced by EGFR-TKI resistance in most cases.
